# Salinity Stress Affects Photosynthesis, Malondialdehyde Formation, and Proline Content in *Portulaca oleracea* L.

**DOI:** 10.3390/plants10050845

**Published:** 2021-04-22

**Authors:** Helena Hnilickova, Kamil Kraus, Pavla Vachova, Frantisek Hnilicka

**Affiliations:** Department of Botany and Plant Physiology, Faculty of Agrobiology, Food and Natural Resources, Czech University of Life Sciences Prague, 165 00 Prague, Czech Republic; krausk@af.czu.cz (K.K.); hnilicka@af.czu.cz (F.H.)

**Keywords:** purslane, salinity, gas exchange, proline, salt stress

## Abstract

In this investigation, the effect of salt stress on *Portulaca oleracea* L. was monitored at salinity levels of 100 and 300 mM NaCl. At a concentration of 100 mM NaCl there was a decrease in stomatal conductance (gs) simultaneously with an increase in CO_2_ assimilation (A) at the beginning of salt exposure (day 3). However, the leaf water potential (ψ_w_), the substomatal concentration of CO_2_ (Ci), the maximum quantum yield of photosystem II (Fv/Fm), and the proline and malondialdehyde (MDA) content remained unchanged. Exposure to 300 mM NaCl caused a decrease in gs from day 3 and a decrease in water potential, CO_2_ assimilation, and Fv/Fm from day 9. There was a large increase in proline content and a significantly higher MDA concentration on days 6 and 9 of salt stress compared to the control group. After 22 days of exposure to 300 mM NaCl, there was a transition from the C4 cycle to crassulacean acid metabolism (CAM), manifested by a rapid increase in substomatal CO_2_ concentration and negative CO_2_ assimilation values. These results document the tolerance of *P. oleracea* to a lower level of salt stress and the possibility of its use in saline localities.

## 1. Introduction

Most crops are sensitive to high salt concentrations in the soil and salinization is one of the most serious environmental factors that can limit crop productivity [1,2]. It has been stated that 20% of the total cultivated and 33% of the irrigated agricultural land worldwide is affected by high salinity and the saline area increases by 10% every year. The yield of most crops is significantly reduced when the electrical conductivity (EC) of the soil reaches 4 dS·m^−1^ (equivalent to 40 mM NaCl) [3]. There are a number of different adaptations that allow certain crop cultivars to continue to grow and produce a harvestable yield under moderate soil salinity [4,5,6].

The adverse effects of salinity are the result of complex interactions among morphological, physiological, and biochemical processes involved in seed germination, plant growth, and water and nutrient uptake [7,8,9]. High concentrations of salt impose both osmotic and ionic stresses on plants [10]. Photosynthesis in all its phases is affected by stress factors, including salinity. The mechanism of photosynthesis involves several components and damage by a stress factor at any level may reduce a plant’s overall photosynthetic capacity [11]. Exposure to salinity leads to closure of the stomata, which acts to limit photosynthesis [12]. Salt-induced osmotic effects can also adversely affect the activities of a number of stomatal enzymes involved in carbon dioxide (CO_2_) reduction [13]. The effects of salinity on photosynthesis may involve inhibition of electron transport and inactivation of the photosystem II (PSII) reaction centers [14], destroying the oxygen-evolving complex (OEC), and impairing the electron transfer capacity on the donor side of PSII [15]. Increased levels of Na^+^ and Cl^−^ in the non-stomatal leaf tissues can also significantly affect metabolic processes that limit photosynthesis [16].

Osmotic stress impairs the ability of plant cells to detoxify reactive oxygen species (ROS). Under normal growth conditions, there is little ROS production in cells. When a plant’s cellular homeostasis is disrupted by some stress factor; however, there is a significant increase in the concentration of ROS [17], producing. 

Harmful effects through lipid peroxidation in cellular membranes, DNA damage, protein denaturation, carbohydrate oxidation, pigment breakdown, and impairment of enzymatic activity [18,19]. 

The preservation of the osmotic gradient through maintaining an appropriate level of compatible osmolytes is also very important [4]. The solutes that accumulate during osmotic changes include amino acids such as proline and quaternary amines like glycine betaine. A dramatic accumulation of proline is a common physiological response in plants exposed to various abiotic stresses [20]. 

The importance of proline lies in its capability of stabilizing proteins, membranes, and subcellular structures, and protecting them from damage by scavenging ROS [21]. Some researchers have shown that there is a higher proline content in saline tolerant genotypes, such as *Solanum tuberosum* L. [22] and *Cucumis melo* L. [23]. De la Torre-Gonzales et al. [24] stated that when *Solanum lycopersicum* L. plants are grown under saline conditions, the magnitude of proline increase corresponded to improvement in its tolerance to salt stress. On the contrary, other authors provided evidence that proline was a stress indicator but did not increase tolerance to salt stress [25,26].

Common purslane (*Portulaca oleracea* L.) is found across the globe and has been used in folk medicine since ancient times. In Chinese medicine *P. oleracea* possesses a wide spectrum of medicinal uses as a neuroprotective, antimicrobial, antidiabetic, antioxidant, and anticancer agent [27]. *P. oleracea* is an annual with good drought and salt tolerance, and its responses to abiotic stresses, such as elevated temperature, drought, and high salinity have been studied [28,29,30,31]. 

Addressing the problems of salinity will be necessary in the future, given the growing population of the planet and the ever-increasing demand for food. At the current rate of world population growth, it will be necessary to produce 70% more food by 2050 than is being produced today [32]. In addition, the changing climatic conditions and their effects on farming threaten the stability of agricultural production. Both research and practice are pursuing a number of ways to address these problems, and improvement in our understanding of the responses of crops to salinity is one of the necessary prerequisites for that. Increased cultivation of halophytic species and their use for phytoremediation could contribute to solving the problem of salinization. The aim of this research was to evaluate the effects of salt stress on the content of free proline and MDA in *Portulaca* and the parameters of gas exchange and chlorophyll fluorescence.

## 2. Results

### 2.1. Effect of NaCl Concentration and Salt Stress Exposure Time on Leaf Water Potential

**Figure 1:**
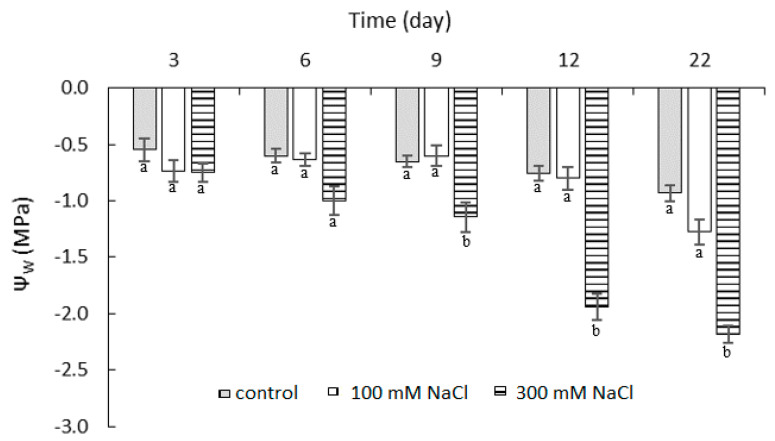
Leaf water potential (ψ_w_) in leaves of *P. oleracea* exposed to 100 mM or 300 mM NaCl. Means ± SE; *n* = 3; one-way ANOVA with Tukey’s post hoc test; *F*_(8, 128)_ = 8.092; *p* = 0.0001. For each time point, the columns with different letters were significantly different (*p* < 0.05).

Salinity reduced the leaf water potential in both experimental treatments (Figure 1). For the 300 mM NaCl treatment, the water potential values ranged from −0.75 to −2.18 MPa. The reduction in leaf water potential was observable from the sixth day of salt stress exposure and was statistically significant relative to control from the ninth day of salt treatment. The water potential decreased rapidly between the 9th and 12th day of salt exposure. For the 100 mM NaCl treatment, the water potential decreased at the end of the experiment, but throughout the experiment, the water potential values were not significantly different from the control.

### 2.2. Effect of NaCl Concentration and Salt Stress Exposure Time on Leaf Gas Exchange Parameters

**Figure 2:**
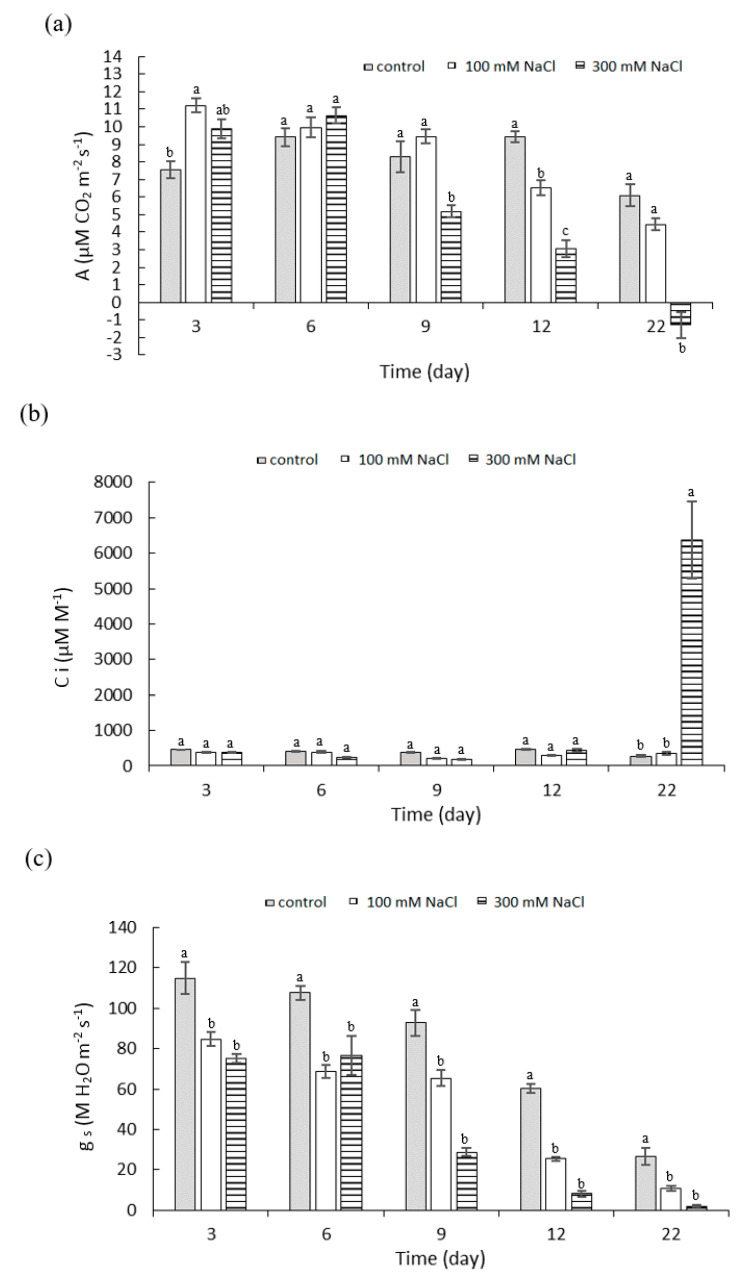
(**a**) Photosynthetic assimilation of CO_2_ (A); (**b**) Substomatal concentration of CO_2_ (Ci) and (**c**) stomatal conductance (gs) of *P. oleracea* under treatment with 100 mM and 300 mM NaCl. Means ± SE; *n* = 3; one-way ANOVA with Tukey’s post hoc test; (**a**) *F*_(8, 207)_ = 44.276; *p* = 0.0001; (**b**) *F*_(8, 207)_ = 44.276; *p* = 0.0001; (**c**) *F*_(8, 207)_ = 44.276; *p* = 0.0001. For each time point, the columns with different letters indicate significant difference at *p* < 0.05.

Changes in CO_2_ assimilation were measured after all treatments (Figure 2a). On the third day of saline stress exposure, the rate of photosynthesis was significantly increased with 100 mM NaCl compared to control, from 7.58 to 11.21 µM CO_2_ m^−2^ s^−1^. A significant decrease in photosynthetic CO_2_ assimilation of 38% under 300 mM NaCl treatment was measured from day 9 of stress, in comparison to the control. In the final stages of the experiment (stress day 22), negative values of CO_2_ assimilation (−1.29 µM CO_2_·m^−2^ s^−1^) were measured. At a salinity of 100 mM NaCl, the CO_2_ assimilation was significantly reduced on stress day 12 compared to control; at the end of the experiment; however, there was no significant difference between control and 100 mM NaCl exposure.

**Figure 3:**
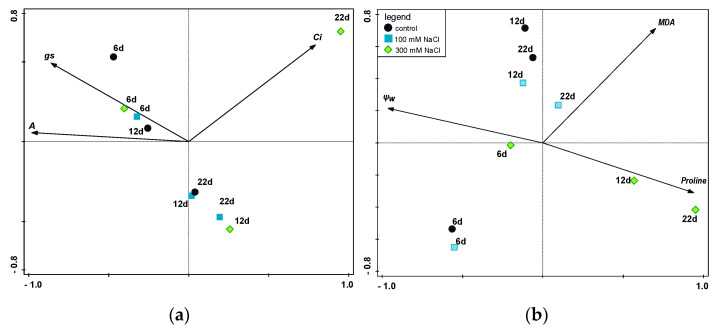
Principal component analysis (PCA) ordination diagram. The biplot displays the treatments (samples) at various times (6 d—day 6, 12 d—day 12, 22 d—day 22) with measurements of (**a**) photosynthetic assimilation of CO_2_ (A), substomatal concentration of CO_2_ (Ci) and stomatal conductance (gs); the first axis accounts for 77.7% of the variance and the second axis shows 20.4% of the variance; (**b**) proline and MDA content in leaves of *P. oleracea* and leaf water potential (ψw); the first axis accounts for 76.8% of the variance and the second axis is responsible for 21.7% of the variance. The treatments are indicated by the colors of the shapes: black circles, control; light blue squares, 100 mM NaCl; green diamonds, 300 mM NaCl.

The values of the substomatal CO_2_ (Ci) concentration at the 100 mM NaCl concentration were stable and comparable to the control throughout the experiment. The same applied to the 300 mM NaCl treatment, with the exception of day 22, when there was a rapid increase in the Ci values (6382.53 µM M^−1^). This increase in the substomatal CO_2_ concentration was almost 23 times higher than the control (Figure 2b). This sharp increase in Ci values was also documented by principle component analysis (PCA) (Figure 3a).

The stomatal conductance was significantly lower than the control for both 100 and 300 mM NaCl, from the third day of stress exposure (84.6 and 75.2 M H_2_O m^−2^ s^−1^, respectively). Differences between the two saline treatments were not significant (Figure 2c). There was a significant decrease in the stomatal conductance values with 300 mM NaCl on days 6 and 9 of stress exposure (76.6 and 28.8 M H_2_O m^−2^ s^−1^, respectively) and at 100 mM NaCl on days 9 and 12 of stress exposure (65.4 and 25.5 M H_2_O m^−2^ s^−1^, respectively). According to the PCA, there was a clear trend of reduction in CO_2_ assimilation over time and also in the dependence on NaCl concentration, where a high salt concentration reduced the CO_2_ assimilation. The stomatal regulation was already noticeable at the beginning of the experiment (Figure 3a). 

### 2.3. Effect of NaCl Concentration and Salt Stress Exposure Time on Chlorophyll Fluorescence

**Figure 4:**
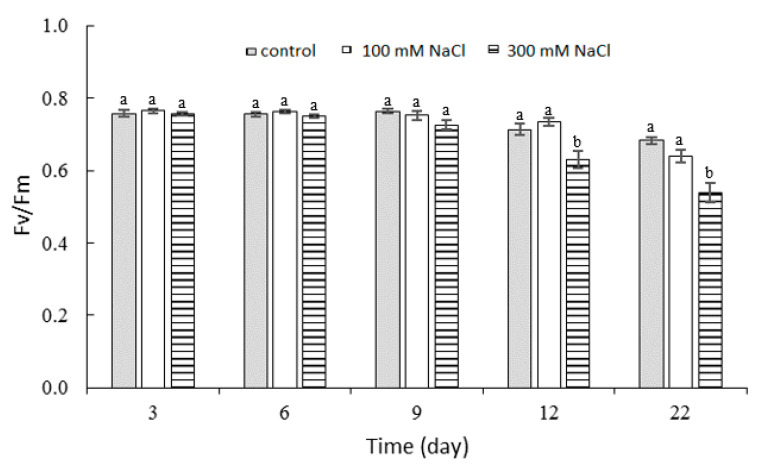
Maximum quantum yield of PS II (Fv/Fm) of *P. oleracea* at different concentrations of NaCl. Means ± SE; *n* = 3; one-way ANOVA with Tukey’s post hoc test; *F*_(10, 223)_ = 3.396; *p* = 0.0001. For each time point, the columns with different letters are significantly different at *p* < 0.05.

For the 100 and 300 mM NaCl treatments, no significant differences were measured in the values of the maximum quantum yield of PSII (Fv/Fm), compared to the control at up to day 9 of stress. A progressive decrease in the maximum quantum yield of the PSII photosystem occurred from day 9 (0.73) until the end of the experiment (0.54) with 300 mM NaCl exposure. Throughout the experiment, there were no significant differences between the control and 100 mM NaCl (Figure 4).

### 2.4. Effect of NaCl Concentration and Salt Stress Exposure Time on Proline and Malondialdehyde Content

**Figure 5:**
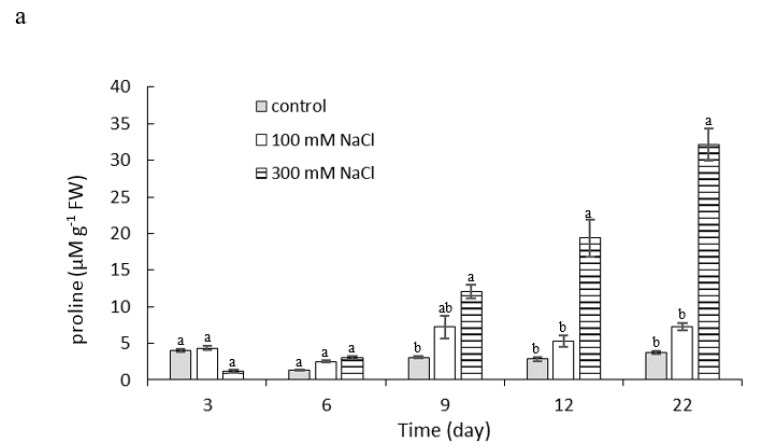

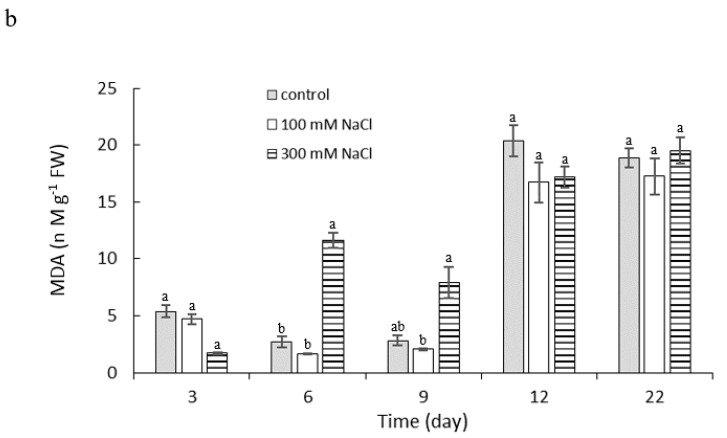
(**a)** Proline and (**b**) MDA content in leaves of *P. oleracea* under salt stress with 100 mM or 300 mM NaCl. Means ± SE; *n* = 3; one-way ANOVA with Tukey’s post hoc test; (**a**) *F*_(8, 207)_ = 44.276; *p* = 0.0001; (**b**) *F*_(12, 81)_ = 6.220; *p* = 0.0001. For each time point, the columns with different letters indicate significant difference at *p* < 0.05.

By the sixth day of salt stress exposure, no significant differences in the free proline content in the leaves of the experimental samples were seen compared to control. A significant progressive increase in free proline content in the leaves was recorded for the 300 mM NaCl treatment from exposure on day 9 until the end of the experiment. The free proline content on day 22 of stress exposure at this salinity was 32.12 µM g^−1^ FW (fresh weight), which is almost 11 times higher than the proline content on day 6 (3.03 µM g^−1^ FW). The content of free proline in the 100 mM NaCl variant ranged from 2.54 to 7.24 µM ·g^−1^ FW throughout the experiment and there was no significant difference relative to control (Figure 5a).

At the initial stages of stress exposure, the differences in MDA level between the salinity treatments were inconclusive. A significantly higher MDA content compared to the control group was seen with the 300 mM NaCl treatment on days 6 and 9 of stress exposure (11.62 and 7.89 nM g^−1^ FW, respectively). For the rest of the experiment, no significant differences between the saline treatments were seen. A common trend for all treatments was an increase in MDA at the end of the experiment (Figure 5b). The PCA results (Figure 3b) showed a close relationship between proline content and high salt concentration, as well as a negative correlation between proline content and water potential (*r* = 0.938).

## 3. Discussion

As carbon dioxide assimilation increases or decreases, the photosynthetic absorption of solar energy, CO_2_ fixation, and glucose formation are likewise altered. The rate of photosynthesis can be affected by environmental factors at all levels, and many researchers have reported a reduction in the rate of photosynthesis due to salinity [3,33,34]. In this study, the plants treated with the lower concentration of salt (100 mM NaCl), showed a higher assimilation of CO_2_ in the initial phases of salt stress (48% more than control), although it is possible to observe a demonstrable decrease in stomatal conductance already in this phase of salt exposure. The stomatal regulation of vapor loss is extremely sensitive to short-term salt stress [35]. Closing of the pores is one of the adaptive mechanisms to prevent loss of cell turgor from limited water supply. The salinity causes a decrease in stomatal conductance, but the rates of photosynthesis per leaf area unit sometimes remain unchanged [3]. In *Arthrocnemum macrostachyum*, which is considered to be extremely well adapted to salinity, an increase in net photosynthesis up to a concentration of 510 mM NaCl has been observed [36]. A reduction of the photosynthesis rate and an increase in intercellular CO_2_ concentration after 14 days of treatment with 100, 150, and 200 mM NaCl was reported by [37]. Tang et al. [38] showed that with increasing NaCl concentration, the intercellular CO_2_ concentration of purslane and the values of chlorophyll a/b increased, while the transpiration rate, net photosynthesis rate, stomatal conductivity and chlorophyll content decreased.

In this study, a significant reduction in CO_2_ assimilation and stomatal conductance with 300 mM NaCl occurred on day 9 of stress exposure. Reducing the stomatal conductance, leads to a reduction in intercellular CO_2_, which decreases the activity of several enzymes including RUBISCO [39], thus limiting carboxylation and reducing the net photosynthetic rate. After 22 days of exposure to 300 mM NaCl, negative values of CO_2_ assimilation and high substomatal CO_2_ concentrations were measured. The *Portulacaceae* is a plant family with remarkable diversity in photosynthetic pathways. This lineage not only has species with different C4 biochemistry (NADP-ME and NAD-ME types) and C3–C4 intermediacy, but also displays different anatomical leaf configurations [40]. *P. oleracea* is characterized by a leaf type with Kranz anatomy and an NAD-malic enzyme (NAD-ME) -type C4 cycle [41]. The genus *Portulaca* contains the only known example of a C4 plant which can switch to crassulacean acid metabolism (CAM) under drought stress [42,43]. Salinity reduced the gas exchange and induced CAM metabolism, thus conferring higher water-use efficiency in *Talinum triangulare* [44].

The CAM gas exchange process has been described in four phases. The nocturnal uptake of CO_2_ via open stomata, fixation by phosphoenolpyruvate carboxylase (PEPC) and vacuolar storage of CO_2_ in the form of organic acids, mainly malic acid comprise phase I. Daytime remobilization of vacuolar organic acids, decarboxylation and refixation plus assimilation of CO_2_ with closed stomata in the Calvin cycle is defined as phase III. Between these two phases there are transitions when the stomata remain open for CO_2_ uptake for a short time during the very early light period (phase II) and reopen again during the late light period for CO_2_ uptake with the direct assimilation to carbohydrate, when the vacuolar organic acid is exhausted (phase IV) [45]. The results obtained at a concentration of 300 mM NaCl in the final phase of the experiment, primarily the negative CO_2_ assimilation, high Ci values, and closed stomata data, can be related to the transition of the C4 cycle of photosynthesis to the CAM cycle. These results correspond with those of Ferrari et al. [46] who stated that CAM was completely reversible and environmentally controlled in *P. oleracea* leaves. An elevated measurement of internal CO_2_ concentration was related to the daily malate decarboxylation (phase III), when the stomatal resistance values were high (stomatal conductivity values were low) [47,48]. High internal CO_2_ concentrations during phase III of CAM and closed stomata allow optimal utilization of light energy [49]. The transition from C4 to CAM under drought conditions and a return to C4 metabolism after subsequent rehydration was reported by [43]. The fixation of CO_2_ in the dark, although representing only 10% of the control plants’ fixation [43] or the reassimilation of CO_2_ from respiration, helped to bridge water deficit periods caused by drought or salinity, and thus helped to maintain a positive CO_2_ balance [50]. The induction of CAM was also demonstrated after 21 to 23 days of drought stress in the C4 plant, *P. oleracea*, in terms of changes in CO_2_ exchange profile, malic acid content and in titratable acidity during the day/night cycle [51].

At the 100 mM NaCl concentration, there was no transition to the CAM cycle, although the net assimilation of CO_2_ and stomatal conductance decreased during the experiment. These parameters also decreased in the control plants. One of the possible causes is leaf senescence, as *P. oleracea* has a brief growing season and the CO_2_ assimilation decreases during senescence, which can be considered a form of stress for the plants [52]. Karkanis and Petropoulos [53] reported a decrease in photosynthesis and stomatal conductance in a set of *P. oleracea* genotypes forty days after sowing and attributed it to exposure to a high temperature (40 °C).

The ratio of Fv/Fm is a very important plant characteristic because it indicates how efficiently the light reaction is proceeding, and it is widely used when studying the impact of stress on plants. An Fv/Fm in the range of 0.79 to 0.84 is a normal value in a number of species [54]. Salt stress of 300 mM NaCl) significantly reduced Fv/Fm on day 12 of exposure. The lower value indicated that a percentage of the PSII reaction centers were damaged or inactivated, a phenomenon commonly observed in plants under stress [55]. Salinity blocked electron transfer from the primary acceptor, plastoquinone (QA) to the secondary acceptor, plastoquinone (QB) at the acceptor side of PSII, which led to a decrease in Fv/Fm [56]. A decrease in the maximum quantum yield of PSII in response to salt was also reported for *Raphanus sativus* L. [57], *Brassica napus* L. [58], and *Eruca sativa* L. [12]. The 100 mM NaCl concentration did not significantly affect Fv/Fm initially compared to the control, but a decrease in the Fv/Fm was observed at both NaCl concentrations in the final phase of the experiment. The ratio of Fv/Fm at 100 mM NaCl was slightly increased compared to 300 mM NaCl [59].

Due to the reduced availability and fixation of CO_2_, an imbalance between electron excitation and utilization by photosynthesis can occur leading to the production of ROS, especially superoxide (O_2_-) and hydrogen peroxide (H_2_O_2_) [60], which can damage cellular structures [61]. ROS detoxification pathways play a protective role in responding to salt stress by removing toxic radicals generated from the mitochondrial and chloroplast electron transport chains. The antioxidant defense systems include both enzymatic (superoxide dismutase, catalase, and ascorbate peroxidase) and non-enzymatic components (carotenoids and glutathione) [62]. In response to salinity, a large number of compatible solutes may accumulate, especially proline. Glycine betaine [63], sugars, and sugar alcohols [64] also play an important role in osmoregulation. A rapid increase in free proline in leaves occurred in the second half of the experiment in plants treated with 300 mM NaCl, while the water potential of their leaves decreased. Proline provides stress protection to the plants by maintaining osmoregulation and detoxifying ROS, which preserves membrane integrity and stabilizes enzymes and other proteins [65,66]. The increase in proline content after 21 days in two *P. oleracea* genotypes, T-16 and WI-9, with 200 mM NaCl salt stress was reported by [67]. Yazici et al. [68] found a threefold increase in proline content in *P. oleracea* plants treated with 140 mM NaCl for 30 days, and stable values for plants treated with 70 mM NaCl for 18 and 30 days. These results are comparable to our data, where plants treated with 100 mM NaCl did not show an increase in free proline content throughout the experiment compared to controls, demonstrating *portulaca’s* tolerance to salinity at this salt level. The increase in free proline in *Portulaca* was reported to be 4.6-fold in comparison with control [53].

Malondialdehyde (MDA) is a natural product of lipid peroxidation and is traditionally used as an indicator of the degree of damage caused by stress to cells [69]. Plants exposed to salinity of 100 mM NaCl did not show a significant increase in lipid peroxidation due to stress compared to control. The common phenomenon in our studied plants was an increased content of MDA due to leaf senescence and an increased rate of lipid peroxidation due to oxidative stress [70]. Similar results were reported by Yazici et al. [68]. In their experiments, the MDA content of the control plants increased by 39% during the trial, but 70 mM NaCl exposure did not increase MDA compared to control. After 30 days of exposure to 140 mM NaCl, however, there was a clear increase in MDA compared to control. Different results were reported by Xing et al. [37] for treatment with 100, 150, and 200 mM NaCl, which increased MDA- and O_2_-production and resulted in damage to cell membrane integrity and protein activity. They stated that long-term exposure to high salinity might destroy some cell membranes, leading to lower SOD, POD, and CAT activities. On the contrary, lipid peroxidation from oxidative stress could not occur within a short period (five weeks) of applied stress [71]. The tolerance of purslane to salinity might be related to an increased capacity of the antioxidant system to scavenge ROS, thus suppressing the level of lipid peroxidation, and inducing the accumulation of osmoprotectant proline under saline conditions [38].

The plants treated with 300 mM NaCl showed an increase in MDA compared to the control groups on days 6 and 9 of the experiment, but in the subsequent phases of stress exposure, the differences were non-significant. An activation of the defense and adaptation mechanisms associated with increased activity of the antioxidant system, limited the formation of ROS, and suppressed the level of lipid peroxidation. These mechanisms are manifested by a rapid increase in proline content and osmotic adaptation, which are closely related to salinity tolerance and the ability of the antioxidant system to scavenge free radicals, suppress lipid peroxidation, and promote the accumulation of osmoprotective agents such as proline.

## 4. Materials and Methods

### 4.1. Experimental Design and Salinity Treatments

The experimental plants of purslane, *Portulaca oleracea* L., cv. Grene, were grown in containers (13 cm × 13 cm) in a garden substrate (pH 5–6.5, nutrient content: N 80–120 mg L^−1^, P 22–44 mg L^−1^, K 83–124 mg L^−1^; 80% white peat, 20% black peat, 20 kg of clay m^−3^; structure 0–10 mm). The plants were grown in the growth room with the following controlled conditions: air temperature of 25 ± 2/18 ± 2 °C day/night, relative humidity 65–75%, light intensity 450 ± 50 μM m^−2^ s^−1^, and photoperiod 12 h light and 12 h dark. Salinity treatments were started on 25-day-old plants with 0 (deionized water, control), 100 and 300 mM NaCl. Measurements of the monitored physiological parameters and the chemical analyses were performed on days 3, 6, 9, 12, and 22 of salt stress.

### 4.2. Leaf Water Potential

Leaf samples for the determination of leaf water potential (ψ_w_; MPa) were placed into a 5 mL syringe, sealed with Parafilm, and frozen at −24 °C. Prior to the measurements, the samples were kept at laboratory temperature until the tissue was completely defrosted. The water potential was determined by putting several drops of the cells sap upon targets of Whatman #1 filter paper (1.5 cm in diameter) and measured using a WP 4C Dew Point PotentiaMeter (Decagon Devices, Inc., Pullman, WA, USA). The measurements were performed with three repetitions of the five plant samples.

### 4.3. Leaf Gas Exchange

The net CO_2_ assimilation (A; μM CO_2_ m^−2^ s^−1^)_,_ stomatal conductance (gs; M H_2_O m^−2^ s^−1^) and substomatal concentration of CO_2_ (Ci; μM M^−1^), were measured in situ when the 4th or 5th fully expanded leaves appeared, using a portable gas exchange system LCi Portable Photosynthesis System (ADC BioScientific Ltd., Hoddesdon, Great Britain). The gas exchange was measured from 9:00 a.m. to 11:00 a.m. The irradiance was 450 μM m^−2^ s^−1^ of photosynthetically active radiation (PAR). With a normal concentration of CO_2_, the temperature in the measurement chamber was 23 °C, and the duration of the measurement of each sample was about 15 min, after the establishment of steady-state conditions inside the measurement chamber. The measurements of these parameters took place on single leaves from three different plants.

### 4.4. Chlorophyll Fluorescence

The minimum chlorophyll fluorescence (F0) and the maximum chlorophyll fluorescence (Fm) were also measured in situ with a portable fluorometer OS5p_+_ (ADC BioScientific Ltd., Hoddesdon, Great Britain) with 1s excitation pulse (660 nm) and a saturation intensity of 3000 μM m^−2^ s^−1^. Measurements were made when the 4th or 5th fully expanded leaves appeared after a 20 min dark adaptation period between 9:00 and 11:00 (local solar time) using leaf-clips which were put on the adaxial leaf blades away from the leaf vein. Dark adaptation time was the time needed to obtain a steady value of Fv/Fm. The maximum quantum yield of PSII was calculated using the formula: Fv/Fm = (Fm − F0)/Fm. These parameters were measured with three repetitions on five plants.

### 4.5. Proline Content

The content of free proline was determined using the method of Bates et al. [72] with modifications. A sample of leaves (0.5 g) was homogenized in 10 mL of 3% sulfosalicylic acid using a mortar and pestle and the homogenate was filtered through filter paper. Aliquots of 1 mL of the filtrate were mixed with 1 mL of acid ninhydrin solution and 1 mL of acetic acid and placed on a shaker for 20 minutes. The samples were then heated at 90 °C for 30 minutes, cooled in ice water, thoroughly mixed with 3 mL of toluene, and incubated 20 min at room temperature. The samples were held for 24 hours at 4 °C, after which the upper layer of the separation mixture was used for measurement of absorbance at 520 nm (UV–Vis, Evolution 210, Thermo Scientific, Waltham, MA, USA). Five plants were used as independent samples for each treatment. The proline concentration was determined using a calibration curve for proline as µM g^−1^ FW (fresh weight).

### 4.6. Malondialdehyde (MDA)

The content of malondialdehyde (MDA) was measured based on a modified thiobarbituric acid (TBA) method [73]. Samples of leaves (0.4 g) were homogenized with liquid nitrogen and 80% ethanol and centrifuged in 2 mL microcentrifuge tubes for 5 min and at 6000 rpm. Aliquots of 0.7 mL of each supernatant were mixed with 0.7 mL of 0.65% TBA in 20% TCA (trichloroacetic acid) and 0.01% BHT (butylated hydroxytoluene) and a second set of 0.7 mL samples was mixed with 0.7 mL 20% TCA and 0.01% BHT. The microcentrifuge tubes were incubated at 95 °C for 25 minutes and after cooling, they were centrifuged for 5 minutes at 6000 rpm. The absorbance at 440 nm, 532 nm, and 600 nm was read on a UV–Vis spectrophotometer (Evolution 210, Thermo Scientific) and the concentration of MDA (nM g^−1^ FW) was calculated using an extinction coefficient of 157 mM cm^−1^.

### 4.7. Statistical Analysis

One-way ANOVA was used to evaluate the variables from the treatments. After obtaining significant results (*p* < 0.05), multiple comparisons using the Tukey HSD test were applied to identify significant differences between treatments. All analyses were performed using Statistica 13.5 software (Statsoft, Tulsa, OK, USA). Program Canoco 5 [74] was used for PCA (principal component analysis). This analysis was appropriate for finding the differences in reactions of *P. oleracea* to the salinity treatments on individual days).

## 5. Conclusions

The results from the combined salinity effects of osmotic and ionic stress were different at different NaCl concentrations manifesting with specific adaptive responses from the plants exposed to stress. The lower salt concentration caused only stomatal regulation of water deficit, as one of the primary regulatory factors of water deficit, without the involvement of other biochemical responses to cope with the stress. At the same time, the lower salt concentration increased photosynthetic CO_2_ assimilation at the beginning of salt exposure. During the stress exposure, the maximum quantum yield of the PSII was not affected, as the Fv/Fm values were comparable to the control sample. The concentration of free proline and MDA was also similar to the control group, meaning that the level of stress did not require an increased proline synthesis for the protection of cellular structures and scavenging ROS. The osmoregulation was not significant at this level of salinity; only after prolonged salt exposure did the water potential of the leaves decrease. The toxic effect of the Na^+^ and Cl^−^ ions and water deficiency at higher NaCl concentrations and a longer exposure elicited a stress response in the form of activation of proline biosynthesis and the accumulation of proline to reduce cellular damage and maintain ROS homeostasis. The limited osmotic availability of water and the necessary stomatal regulation, together with the need for a supply of CO_2_, caused the transition from the C4 CO_2_ fixation mechanism to the CAM mechanism. This salinity tolerance allows *Portulaca oleracea* to be cultivated in slightly saline localities, or to be potentially used for other technological measures, such as co-cultivation with sensitive glycophytic species. Given the current knowledge about the behavior of *Portulacaceae* under saline conditions, further research in this area is recommended.

## Data Availability

Data is contained within the article.
